# Screening of germline mutations in young Rwandan patients with breast cancers

**DOI:** 10.1002/mgg3.1500

**Published:** 2020-09-22

**Authors:** Jeanne P. Uyisenga, Karin Segers, Aimé Z. Lumaka, Pacifique Mugenzi, Corinne Fasquelle, Bouchra Boujemila, Claire Josse, Leon Mutesa, Vincent Bours

**Affiliations:** ^1^ Laboratory of Human Genetics GIGA Research Institute University of Liège Liège Belgium; ^2^ Department of Biology College of Science and Technology University of Rwanda Kigali Rwanda; ^3^ Department of Human Genetics University Hospital of Liège CHU Liège Liège Belgium; ^4^ Rwanda Military Hospital Kigali Rwanda; ^5^ Department of Medical Oncology University Hospital of Liège CHU Liège Liège Belgium; ^6^ Center for Human Genetics College of Medicine and Health Sciences University of Rwanda Kigali Rwanda

## Abstract

**Background:**

In Sub‐Saharan Africa breast cancer is commonly detected at younger age and the profile is more aggressive with a high mortality rate compared to the European countries. It is suggested that African‐specific genetic background plays a key role in this matter. The present study aimed at understanding the role of genetic factors in breast cancer development in young Rwandan.

**Methods:**

We performed a massive parallel sequencing on Illumina MiSeq NGS system for the screening of 26 genes associated with hereditary breast cancer from 40 patients under 35 years old from two University Teaching Hospitals in Kigali, Rwanda. Sanger sequencing was used to confirm pathogenic and likely pathogenic mutations.

**Results:**

Five patients out of 40 (12.5%) presented with pathogenic mutations including four patients (10%) carrying *BRCA1* or *BRCA2* pathogenic variants. One patient showed a missense likely pathogenic *TP53* variant. We have also detected additional missense, intronic, and 3’UTR variants of unknown significance in all study participants.

**Conclusion:**

This preliminary study suggests that the frequency of germline mutations in young Rwandan patients with breast cancer is similar to the observations made in Caucasians. However, further large studies including patients and controls are needed to better understand the impact of genetic factors as well as the environmental risk factors in the development of breast cancer in young Rwandans.

## Abstract 2

Screening of germline mutations in Rwandan young patients with breast cancer. The next generation sequencing detected pathogenic BRCA 1 and 2 mutations in 6 patients. We have also detected additional missense, intronic, and 3’UTR variants of unknown significance in all study participants.

## 1 INTRODUCTION

Breast cancer (BC) is a common cause of mortality among women worldwide. In individuals under 40 years old, it is however considered to be rare as it affects less than 7% of patients (Brinton, Sherman, Carreon, & Anderson, 2008).

Although its incidence in African ancestry individuals is still lower compared to other ethnic groups, the mortality is rather higher. Indeed, breast cancer in sub‐Saharan Africa is characterized by a younger age at diagnosis (Adebamowo et al., 2003; Fregene et al., 2005; Adesunkanmi, Lawal, Adelusola, & Durosimi, 2006). Compared to other age groups, BC in young people (YBC) has a worse prognosis due its advanced stage at diagnosis and a high proportion of hormone negativity subtype (Anders et al., 2008; Bharat, Aft, Gao, & Margenthaler, 2009; Colzani et al., 2011).

About 5%–10% of BC are caused by germline mutations. To date, inherited mutations associated with breast cancer risk have been identified in several genes. Those genes have been associated with different levels of risk of breast cancer ranging from high, moderate to low risk. High‐risk genes include *BRCA1* (OMIM: 113705), *BRCA2* (OMIM: 600185), and *TP53* (OMIM: 191170) and confer a lifetime relative risk of more than five. *BRCA1* and *BRCA2* are two major genes associated with a lifetime risk of 50%–80% of breast cancer. Other genes have been associated with a two to fivefold increase risk of breast cancer. These include genes that are involved in DNA breaks repair by homologous recombination such as *PALB2* (OMIM: 610355), *ATM* (OMIM: 607585), and *CHEK2* (OMIM: 604373; Wittersheim, Büttner, & Markiefka, 2015).

The cause of breast cancer associated with a high mortality rate in young African are still not well understood and remain understudied. A common hypothesis is that the YBC may be linked to African‐specific genetic characteristics (Haffty et al., 2009; Rummel, Lovejoy, Shriver, & Ellsworth, 2017).

Few studies have been conducted in Africa to determine the role of genetic factors in development of BC in general, and in young patients in particular. The majority of those studies restricted their investigations to the screening of mutations in *BRCA1* and *BRCA2* genes (Abbad et al., 2018).

In Rwanda, genetic risk factors, incidence, and mortality rate of BC are not known. A recent study conducted at Butaro cancer center of excellence, it was reported that the median age at diagnosis of breast cancer was 49 and 32/144(22%) patients were below the age of 40 (Pace et al., 2015).

The determination of genetic variations associated with the occurrence of BC as well as genetic modifiers leading to the disease variability are necessary for accurate detection, prevention, and treatment.

We undertook this study to determine the germline mutations associated with BC disease in young Rwandan patients.

## 2 MATERIALS AND METHODS

### 2.1 Ethical compliance

This study was conducted in accordance with the Declaration of Helsinki and the approval of the Institutional Review Board (IRB) of the College of Medicine and Health Sciences (CMHS) at University of Rwanda (No: 156/CMHS IRB/2016) as well as the ethical committee of each of hospitals: Kigali University Teaching Hospital (CHUK) Clinical Research Ethical Committee (Ref.: EC/CHUK/089/2016) and Rwanda Military Hospital (RMH) Research Ethical Committee (Ref.: EC/RMH/051/2016).

All adult patients and parents of minor patients (under 21 years old) signed a written informed consent prior to enrollment.

### 2.2 Study participants

Patients were recruited between April 2016 and March 2018 from the two main public Hospitals in Kigali CHUK and RMH. These two hospitals receive patients from all parts of the country. To be eligible for this study, patients had to be diagnosed with BC before the age of 35. Forty patients consented to participate in the study. Clinical information as well as data on family history of cancer were collected from patient's medical records. Venous blood samples were collected during their routine hospital visits.

### 2.3 DNA extraction

Whole blood samples were collected in EDTA tubes and stored at –20°C until use. Genomic DNA was extracted from 200 μL of whole blood samples using the QIAamp^®^ Blood DNA Mini Kit (Qiagen) according to the manufacturer's manual. The quality of the isolated DNA was assessed using NanoDrop Spectrophotometry ensuring the ratio of A_260 nm_/A_280 nm_ ≈ 2.

### 2.4 Next‐generation sequencing and variant call

Libraries for next‐generation DNA sequencing were constructed from **75 **ng of the isolated DNA for each sample using BRCA Hereditary Cancer MASTR Plus kit (BRCA HC MASTR Plus; MR‐0320.024, Agilent) following the manufacturer's instructions. This kit is designed for the identification of single nucleotide variants (SNVs), insertions and deletions (indels), and copy number variations (CNVs) within the 26 following genes: *BRCA1*, *BRCA2*, *CHEK2*, *BARD1* (OMIM: 601595), *BRIP1* (OMIM: 605882), *RAD51C* (OMIM: 602774), *RAD51D* (OMIM: 602954), *TP53*, *MRE11A* (OMIM: 600814), *RAD50* (OMIM: 604040), *NBN* (OMIM: 602667), *FAM175A* (OMIM: 611143), *ATM*, *PALB2*, *STK11* (OMIM: 602216), *MEN1* (OMIM: 131100), *PTEN* (OMIM: 601728), *CDH1* (OMIM: 192090), *MUTYH* (OMIM: 604933), *BLM* (OMIM: 210900), *XRCC2* (OMIM: 600375), *MLH1* (OMIM: 20436), *MSH6* (OMIM: 600678), *PMS2* (OMIM: 600259), *MSH2* (OMIM: 609309), and the *3’UTR* of *EPCAM* (OMIM: 185535). The same manufacturer also provides a bioinformatics platform, MASTR Reporter for automated quality control and data analysis.

Briefly, targeted DNA regions made up by all coding exons and flanking intronic regions of the 26 genes, were first amplified in multiplex polymerase chain reaction (PCR)––five plexes per patient––using BRCA HC MASTR Plus (Agilent), then barcoded with specific molecular identifiers (MIDs) and ligated with adaptors using MID Dx (MID ML‐2208.240) for Illumina Miseq^®^ sequencer following the manufacturer's instructions. Amplicons were pooled together and purified using AMPure XP Agencourt beads (Beckman Coulter Inc, USA) prior to sequencing. Sequencing was performed on Illumina Miseq NGS system using MiSeq Reagent Kit v3 (paired‐end; 600‐cycles; 2x 300 +++pb; MS‐102‐3003‐Illumina) according to the manufacturer's instructions. Data were analyzed using MASTR reporter software v 1.0.2 (Agilent) and verified using SeqPilot (Module SeqNext) software v.4.3.1. Both softwares align to *hg*19 reference genome: *BRCA1* (NM_007294.4), *BRCA2* (NM_000059.4), *CHEK2* (NM_001005735.2), *BARD1* (NM_000465.4), *BRIP1* (NM_032043.3), *RAD51C* (NM_058216.3), *RAD51D* (NM_001142571.2), *TP53* (NM_000546.6), *MRE11A* (NM_005591.4), *RAD50* (NM_005732.4), *NBN* (NM_001024688.3), *FAM175A* (NM_139076.3), *ATM* (NM_000051.4), *PALB2* (NM_024675.4), *STK11* (NM_000455.5), *MEN1* (NM_130802.2), *PTEN* (NM_000314.8), *CDH1* (NM_004360.5), *MUTYH* (NM_001128425.2), *BLM* (NM_001287248.2), *XRCC2* (NM_005431.2), *MLH1* (NM_000249.4), *MSH6* (NM_000179.3), *PMS2* (NM_000535.7), *MSH2*: (NM_000251.3), and *EPCAM(3’UTR)*; NM_002354.3). At least 98% of all amplicons were covered with a minimum depth of coverage of 40x. For each retained variant, the MASTR reporter software provided annotations including its genomic position, the nucleotide change and the predicted protein change. The impact and consequence on the gene product were predicted by the Ensembl Variant Effect Predictor (VEP) tool v.83.

Sanger sequencing on ABI 3130 using standard dideoxy termination procedure was performed for the validation of plausible pathogenic variants.

### 2.5 Variants classification and report

As there is no specific database for mutations in African population, the pathogenicity of variants was evaluated using two mutation databases: ClinVar ( www.clini​var.com) and dbSNPs ( www.ncbi.nlm.nih.gov/snp). The pathogenicity effect of the VUS were evaluated using different *in silico* mutation interpretation softwares such as SIFT (Sorting intolerant form tolerant); http://sift-dna.org; which predicts where an amino acid substitution is deleterious to protein function; PROVEAN (Protein variation effect analyzer) www.//provean.jcvi.org which predicts the functional effect on protein sequence variations; and Mutation Taster www.mutti​ontas​ter.org which evaluate the disease‐causing potential of DNA variants sequences.

Variants were classified into pathogenic, likely pathogenic, VUS, or likely benign/benign following the recommendation of Association of Molecular Pathology, American Society of clinical Oncology, and College of American Pathologists (AMP‐ASCO‐CAP; Li et al., 2017) and annotated according to the Human Genome Variation Society (HGVS) recommendations (den Dunnen et al., 2016).

## 3 RESULTS

### 3.1 Clinical characteristics of patients

Forty Rwandan breast cancer patients were enrolled in this study. They were selected based on the early age onset independently of family history of cancer. The mean age at diagnosis was 31.2 ± 3.6 years; ranging from 17 to 34 years. Histologically, 33 patients (82.5%) presented with invasive ductal carcinoma (IDC) tumors. Invasive carcinoma of both ductal and lobular and a sarcoma of the breast were present in one and two patients, respectively. Twenty‐one patients (52.5%) were at stage three of tumor development. Twenty‐three patients in our cohort (58.9%) had ER negative tumors and 14 (38.9%) had Her2 positive tumors. Seven patients (17.5%) had a triple negative subtype. Nine patients (22.5%) had a family history with the first or second‐degree relatives (FDR or SDR) with ovarian or breast cancer (HBOC). The characteristics of patients, family history, and others risk factors are summarized in Table 1.

**TABLE 1:** Characteristics of 40 young patients.

N = 40			
Age at diagnostic	Mean±SD		31.15 ± 3.6
Age at first menarche			14 ± 1.56
Parity			2.13 + 1.42
		Number (n)	%
Laterality	Left	18	45.0%
	Right	21	52.5%
	Bilateral	1	2.5%
Histology	IDC	33	82.5%
	ILC	2	5.0%
	IDC&ILC	1	2.5%
	Sarcoma	2	5.0%
	Metaplastic	1	2.5%
	Phyllodes	1	2.5%
Stage	I	1	2.5%
	II	15	37.5%
	III	21	52.5%
	IV	1	2.5%
Lymph nodes involvement	Yes	24	64.86%
	No	13	35.14%
	Missing	3	
ER status	ER‐	23	58.97%
	ER+	16	41.03%
	Missing	1	
PR status	PR‐	13	92.86%
	PR+	1	7.14%
	Missing	26	
Her2 status	Her2‐	22	61.1%
	Her2+	14	38.9%
	Missing	4	
Triple negative subtype (TN)		7	17.5%
FDR /SDR with HBOC		7	27%
FDR /SDR with another type of cancer		3	12%
FDR /SDR with both HBOC and another type cancer		2	8%
No familial history of cancer		14	54%

Abbreviations: DCIS, Ductal carcinoma *in situ*; ER, Estrogen receptor; FDR, First degree relative; HBOC, History of breast and ovarian cancer; Her2, human growth factor 2; *IDC*, invasive ductal carcinoma; ILC, Invasive lobular carcinoma; Med, median; P, Percentile; SDR, second degree relative.

### 3.2 Mutational status

#### 3.2.1 Pathogenic or likely pathogenic variants.

Data analysis revealed plausible pathogenic variants in five patients among 40 participants (12.5%) in three genes (*BRCA1*, *BRCA2*, and *TP53*) out of 26 contained in the panel. Four patients (10%) carried *BRCA1* or *BRCA2* pathogenic mutations: one had a *BRCA2*:c.1300_1303del p.(Lys434Glufs*25) mutation, the second had a *BRCA2*:c. 3720_3723del p.(Phe1241Valfs*17) mutation, the third had a *BRCA*2:c.9097dupA p.(Thr3033Asnfs*11) while the fourth carried a *BRCA1*:c.4065_4068del p.(Asn1355Lysfs*10) mutation. A fifth patient had a missense likely pathogenic *TP53*: c.726C>G (p.Cys242Trp) mutation (Table 2). Sanger sequencing confirmed the five mutations (Supplementary file S1: mutation *BRCA2*:c.1300_1303del; Supplementary file S2: mutation *BRCA2*:c. 3720_3723del; Supplementary file S3: mutation *BRCA1*:c.4065_4068del; Supplementary file S4: mutation *TP53*: c.726C>G and Supplementary file S5: mutation *BRCA*2:c.9097dupA).

**TABLE 2:** Five pathogenic variants identified in 40 young Rwandan patients.

Patient ID	Patient age	Tumor stage	Tumor subtype	Family history	Gene	Nucleotide change	Protein effect	Coding impact	rsID
BC01	27	II	ER+PR+Her2‐	Two Aunts, 38 and 46 years, BC	*BRCA2* a	c.1300_1303del	p.(Lys434Glufs*25)	Frameshift	rs397507577
BC05	34	III	ER+Her2‐	Sister, 42 years, BC	*BRCA2*	c.3720_3723del	p.(Phe1241Valfs*17)	Frameshift	rs886038093
BC40	34	III	ER‐PR‐Her2+	Unknown	*BRCA2*	C.9097dupA	p.(Thr3033Asnfs*11)	Frameshift	rs397507419
BC22	33	II	ER‐PR‐Her2‐	Unknown	*BRCA1* b	C.4065_4068del	p.(Asn1355Lysfs*10)	Frameshift	rs80357508
BC23	31	III	ER+Her2+	unknown	*TP53* c	c.726C>G	p.Cys242Trp	Missense	rs375874539

a Reference sequence BRCA2 (NM_000059.4).

b Reference sequence: BRCA1 (NM_007294.4).

c Reference sequence: TP53 (NM_000546.6).

#### 3.2.2 Variants with unknown significance (VUS)

In total, 33 VUS were identified. Each patient carried at least one VUS and five patients had more than two VUS in the same or different genes. Twenty‐nine VUS among them (87.8%) were unique in this cohort. Eight of those VUS were predicted by different prediction tools (SIFT, Provean, and MutationTaster) to have a damaging effect on protein and 13 variants were very rare and not previously observed in African population while five variants were simply novel (Table 3).

**TABLE 3:** Thirty‐three Variants of unknown significance (VUS) identified in 40 young Rwandan patients.

Gene	Reference sequence	Variant	Protein effect	Coding impact	rsID	No. carriers	GMAF	MAF—Africa
*ATM*	NM_000051.4	*c.4339A>C*	*p.(Ser1447Arg)*	*missense*	*NA*	*1*	*NA*	*NA*
*ATM*	NM_000051.4	c.2289 T > A	p.(Phe763Leu)	missense	rs34231402	1	0.0005	0.002
*ATM*	NM_000051.4	c.131A>G^a^	p.(Asp44Gly)	missense	rs150143957	1	0.00003	0.0005
***BARD1***	NM_000465.4	**c.1148 T > A**	**p.(Met383Lys)**	**missense**	**rs763596413**	**1**	**0.000008**	**0**
*BARD1*	NM_000465.4	*c.421C>T*	*p.(Pro281Leu)*	*missense*	*NA*	*1*	*NA*	*NA*
*BLM*	NM_001287248.2	c.3879A>G	p.(Glu1293=)	synonymous	rs28377085	1	0.00031	0.00314
*BLM*	NM_001287248.2	c.1881 T > C	p.Thr627	synonymous	rs148678729	1	0.00003	0.0002
*BRCA1*	NM_007294.4	c.5411 T > C	p.(Met1804 Thr)	missense	rs55808233	1	0.0002	0.0018
***BRCA1***	NM_007294.4	**c.−16A>G**		**5'UTR Substitution**	**rs777262055**	**1**	**1.00E−05**	**3.00E−05**
*BRCA2*	NM_000059.4	*c.7502A>G*	*p.(Gln2501Arg)*	*missense*	*NA*	*1*	*NA*	*NA*
*BRIP1*	NM_032043.3	c.778A>G	p.(Thr260Ala)	missense	rs138743097	1	0.0004	0.002
*BRIP1*	NM_032043.3	c.854A>G^a^	p.(His285Arg)	missense	rs141055990	1	0.0004	0.0002
***CDH1***	NM_004360.5	**c.1004G>A^a^**	**p.(Arg335Gln)**	**missense**	**rs373364873**	**1**	**0.00003**	**NA**
*CHEK2*	NM_001005735.2	*c.1298A>G^a^*	*p.(Tyr433Cys)*	*missense*	*rs200928781*	*11*	*0.0000*	*NA*
***CHEK2***	NM_001005735.2	**c.1270A>G^a^**	**p.(Met424Val)**	**missense**	**rs375130261**	**3**	**0.00003**	**NA**
*EPCAM* (3’UTR)	NM_002354.3	c.78A>T		3'UTR substitution	rs568965134	1	0.0002	0.001
***MLH1***	NM_000249.4	**c.380+16C>G**		**intronic**	**rs121909452**	**1**	**NA**	**NA**
***MLH1***	NM_000249.4	**c.1730C>T^a^**	**p.(Ser577Leu)**	**missense**	**rs56185292**	**1**	**0.00006**	**0**
***MRE11A***	NM_005591.4	**c.256G>A^a^**	**p.(Asp86Asn)**	**missense**	**rs763902512**	**1**	**0.00001**	**0.00002**
*MRE11A*	NM_005591.4	c.2080‐23A>G		intronic substitution	rs142331797	1	0.0008	0.0002
*MSH2*	NM_000251.3	c•1C>G		3'UTR substitution	rs114545543	1	0.0004	0.0009
*MSH2*	NM_000251.3	*c.301G>C*	*p.(Glu101Gln)*	*missense*	*NA*	*1*	*NA*	*NA*
***MUTYH***	NM_001128425.2	**c.217G>A**	**p.(Glu73Lys)**	**missense**	**rs1064794128**	**1**	**NA**	**NA**
***NBN***	NM_001024688.3	**c.1711A>G**	**p.(Lys571Glu)**	**missense**	**rs587780090**	**1**	**0.00001**	**0**
*NBN*	NM_001024688.3	c.1354A>C	p.(Thr452Pro)	missense	rs141137543	1	0.0004	0.0009
*PMS2*	NM_000535.7	c.924G>C	p.(Glu308Asp)	missense	rs114185660	1	0.0004	0.001
***PMS2***	NM_000535.7	**c.1004A>G^a^**	**p.(Asn335Ser)**	**missense**	**rs200513014**	**1**	**0.00004**	**0**
*PMS2*	NM_000535.7	c.2350G>A	p.(Asp784Asn)	missense	rs143340522	3	0.0013	0.00695
***PMS2***	NM_000535.7	**c.130_131delinsCT**	**p.(Leu458Ser)**	**missense**	**rs587778615**	**10**	**NA**	**NA**
*RAD51C*	NM_058216.3	*c.965+28C>T*		*intronic substitution*	*NA*	*1*	*NA*	*NA*
*RAD51D*	NM_001142571.2	c.322C>T	p.(Arg108Cys)	missense	rs142387263	1	0.0004	0.001
***TP53***	NM_000546.6	**c.993+165_993+166dup**		**intronic insertion**	**rs775788764**	**1**	**0**	**0**
***XRCC2***	NM_005431.2	c.*3 T > C		**3'UTR Substitution**	**rs754786665**	**1**	**8.89E−06**	**0**

Note In bold: Very rare variants (with GMAF ≈ 0); In italic: Novel variants;^a^ Variants predicted by SIFT, Provean, Polyphen‐2 and MutationTaster to be damaging on the protein.

## 4 DISCUSSION

In the present study, we have sequenced genomic DNA from 40 Rwandan patients aged below 35 at the time of breast cancer diagnostic. Young age at diagnosis and African origin are both well known to be associated with an advanced stage of the disease at diagnostic, a high proportion of hormone receptor negative tumors, and a worse prognosis. The reasons of this different severity when compared to Caucasian populations are still poorly understood but are thought to be related to African‐specific genetic characteristics (Adesunkanmi et al., 2006), and/or environmental factors (Fregene et al., 2005). We conducted this study to gain insights into relevant genetic variations in young Rwandan with breast cancer.

We observed an overall frequency of 5/40 (12.5%) pathogenic germline variants. Among them, 10% variants were detected in *BRCA1* and *BRCA2*. This frequency was comparable with other high frequencies of pathogenic *BRCA1*/*2* variants reported in other studies conducted in young Africans (Awadelkarim et al., 2007; Cherbal et al., 2010), Caucasians (Copson et al., 2018; De Sanjosé et al., 2003; Tonin et al., 2001), or African American women with breast cancers (Haffty et al., 2009; Malone et al., 2006; Table 4). The four pathogenic variants in *BRCA1* and *BRCA2* were well known in other populations (Heramb et al., 2018), or reported by ENIGMA.

**TABLE 4:** *BRCA1*/*2* mutations frequencies in young women of Caucasians, African American, and Africans.

Study	Mutation frequencies	Sample size (n)	Age limit	Country
Africans				
This study	10.0%	40	<35 years old	Rwanda
Francies et al., (2015)	7.7%	78	<50 years old	South Africa (Francies et al., 2015)
Fackenthal et al., (2012)	11.0%	265	<50 years old	Nigeria (Ibadan; Fackenthal et al., 2012)
Tazzite et al., (2012)	12.5%	72	<50 years old	Morocco (Tazzite et al., 2012)
Cherbal et al., (2010)	14.0%	49	≤ 40 years old	Algeria (Cherbal et al., 2010)
Troudi et al., (2007)	18.0%	36	≤ 40 years old	Tunisia (Troudi et al., 2007)
Awadelkarim et al., (2007)	12.0%	34	≤ 40 years old	Sudan (Awadelkarim et al., 2007)
Fackenthal et al., (2005)	2.5%	39	≤ 40 years old	Nigeria (Fackenthal et al., 2005)
Gao et al., (2000)	4.0%	70	≤ 40 years old	Nigeria (Gao et al., 2000)
Caucasians				
Copson et al., (2018 **)**	12.0%	2733	≤ 40 years old	UK (Copson et al., 2018)
de Sanjosé et al., (2003)	11.6%	136	≤ 40 years old	Spain (De Sanjosé et al., 2003)
Tonin et al., (2001)	13.0%	61	≤ 40 years old	Canada (Montreal; Tonin et al., 2001)
African American				
Haffty et al. (2009)	14.0%	39	<45 years	USA (New Jersey; Haffty et al., 2009)
John et al. (2007)	17.0%	30	<35 years old	USA (North California; John, et al., 2007)
Malone et al., (2006)	10.3%	80	<45 years	USA (Seattle; Malone et al., 2006)

https://clinv​armin​er.genet​ics.utah.edu/varia​nts-by-submi​tter/50486​3/gene/BRCA2/​patho​genic. In Africa, the pathogenic variant *BRCA1*: c.4065_4068del, observed in one patient of our cohort (33 aged), was previously observed in a 38‐aged Algerian (Cherbal et al., 2010) and a 28‐aged Sudanese (Awadelkarim et al., 2007) breast cancer patients. The patient of our cohort had a triple negative (TN) breast cancer subtype.

We identified less *BRCA1* pathogenic variants compared to *BRCA2* (25%vs.75%) in this cohort. This is in consistence with results from other studies on breast cancer in patients of African ancestry where plausible causal variants in *BRCA2* gene were predominant compared to *BRCA1* (Gao et al., 2000; Panguluri et al., 1999). Contrarily, studies in Caucasians (Krainer et al., 1997) report more *BRCA1* mutations in early onset breast cancers than *BRCA2*. Unfortunately, we cannot make a final conclusion because of a small sample size of our cohort.

In our cohort, only one patient (2.5%) harbored a likely pathogenic *TP53*: c.726C>G variant. This result is similar to previous studies (Bougeard et al., 2015; Hauke et al., 2018), where germline pathogenic *TP53* mutations were found in up to 5% of young breast cancer patients.

Surprisingly, in the youngest patient in our cohort, who had familial history of breast cancer and presented with a sarcoma of the breast cancer and a TN tumor, did not have a plausible *TP53* or *BRCA1*/*2* variants, as one would expect. She did not harbor a plausible variant in the other genes of the tested panel neither.

A high number of VUS (n = 33) was observed in our study; which is in consistent with other studies in black women of African ancestry where NGS panel were evaluated (Awadelkarim et al., 2007; Fackenthal et al., 2012). These variants may have no functional implication in hereditary breast cancer, but their clinical significance remains to be elucidated. Our analysis indicated that eight among these VUS were predicted by SIFT, Provean, and Mutation Taster to have a damaging effect on protein (Table 3). Those predicted damaging VUS include one variant in *CDH1* and *BRIP1* genes, respectively, and two variants in each of the following three genes: *ATM*, *CHEK2*, and *PMS2*. The four genes namely *CDHI*, *BRIP1*, *ATM*, and *CHEK2* are known to be associated with a high or moderate risk of breast cancer. However, the association of *PMS2* gene with breast cancer is still unclear. In Caucasians, contradictory reports were published on the association of *PMS2* gene mutations in breast cancer (Bernstein et al., 2019), (Roberts et al., 2018). The impact of this gene in the development of breast cancer in Africa needs further investigation.

Some variants identified in this study were classified as VUS because they are not, at the time of the redaction of this manuscript, found in mutation databases or clinical reports. Thus, the final frequency of germline mutations in our cohort is pending upon further evidences and reports from the literature and databases.

We found 13 variants that had never been observed before in African population (Ensembl: GMAF=0) and five variants that had never been observed before in any population. The lack of African reference and diseases databases are still preventing the full interpretation of NGS data from African individuals. This causes possible underestimation of the role of germline mutations in development of genetic diseases such as breast cancer in African population in general and in young Rwandan in particular. The development of such databases will allow more reliable determination of genetic contribution to breast cancer development in young Africans.

This study is among very few cohort‐based studies in Sub‐Saharan Africa investigating the contribution of germline mutations to breast cancer within a large panel of breast cancer susceptibility genes. The majority of previous studies conducted in Africa were limited to the assessment of mutations in *BRCA1* and *BRCA2* only. However, we did not detect any clear relevant variant in 23 genes out of 26, indicating that *BRCA1* and *BRCA2* are probably the most commonly mutated genes associated with breast cancer predisposition in African women.

Our study had limitations related to the small size of our cohort, as well as the lack of reference mutation database for African population. These may lead to a false estimation of the frequency of genetic mutations. Additionally, we have only sequenced the coding sequences and their flanking intronic regions, and interrogated SNVs and small Indels. We may have missed the deep intronic variants or CNVs that would be associated with a risk of breast cancer.

## 5 CONCLUSION

Our preliminary results showed that in young Rwandan patients with breast cancers, *BRCA* genes were the most mutated with a predominance of *BRCA2* variants. The frequency of overall mutations was similar to the results observed in Caucasians. Further large studies including both large families and controls and interrogating more types and locations of variants would be interesting to better understand the impact of germline mutations and environmental risk factors in the development of breast cancer in young Rwandans.

## CONFLICT OF INTEREST

The authors declare that they have no conflict of interest.

## AUTHOR’S CONTRIBUTION

J.P.U, V.B, and L.M conceived the study; J.P.U, C.J, K.S, P.M, B.B, and C.F performed all laboratory tests and analyses; J.P.U, K.S, and A. Z. L. analyzed and interpreted results; V.B and L.M supervised the study; J.P.U wrote the paper with the contribution of all authors; All authors reviewed the manuscript and approved the final manuscript's content.

## Supporting information

Supplementary Material

## Data Availability

The data generated and/or analyzed during the current study are available from the corresponding authors on reasonable request.
