# Supplementary material for: Screening of germline mutations in young Rwandan patients with breast cancers
Source: Mol Genet Genomic Med. 2020 Sep 22;8(11):e1500. doi: 10.1002/mgg3.1500 (PMC7667342; doi:10.1002/mgg3.1500)

**SUPPLEMENTARY FILE S1**

**Title**: Screening of germline mutations in young Rwandan patients with breast cancers.

**Molecular Genetics and Genomic Medicine Journal**

**Authors**

Jeanne P. Uyisenga^1,2^, Karin Segers^3^, Aimé Z. Lumaka^1^, Pacifique Mugenzi^4^, Corinne Fasquelle^1^, Bouchra Boujemila^1^, Claire Josse^1,5^, Leon Mutesa^6^ and Vincent Bours^1,3^

**Affiliations**

^1^Laboratory of Human Genetics, GIGA Research Institute, University of Liège, B34, +2, 4000 Liège, Belgium

^2^Department of Biology, College of Science and Technology, University of Rwanda, 4285 Kigali, Rwanda.

^3^Department of Human Genetics, University Hospital of Liège (CHU Liège), B23, +2, 4000 Liège, Belgium.

^4^Rwanda Military Hospital, Kanombe, 4062 Kigali, Rwanda.

^5^Department of Medical Oncology, University Hospital of Liège (CHU Liège), B35, -2, 4000Liège, Belgium.

^6^ Center for Human Genetics, College of Medicine and Health Sciences, University of Rwanda, KK11 Ave. Remera Campus, Kigali, Rwanda.

Correspondence to Prof Leon Mutesa: [lmutesa@gmail.com](mailto:lmutesa@gmail.com) ; Prof. Vincent Bours: [v.bours@uliege.be](mailto:v.bours@uliege.be)

**Supplementary data S1. Confirmation of pathogenic mutation** ***BRCA2* :c.3720_3723del by Sanger sequencing**

Patient BC05


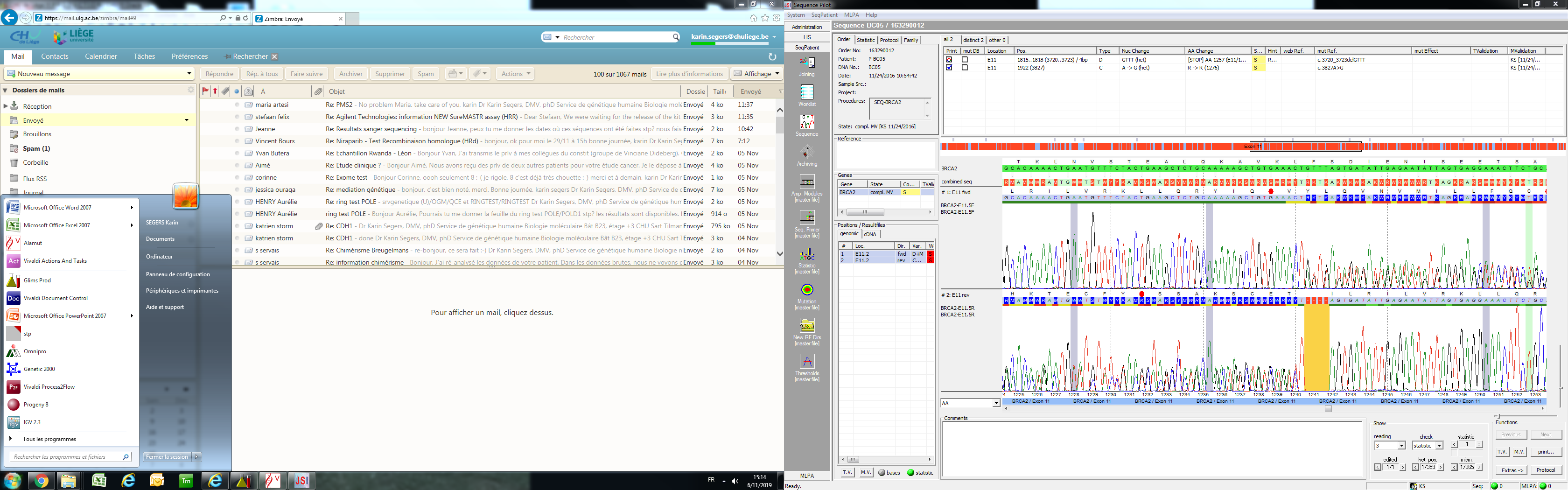


**Supplementary data S2. Confirmation of pathogenic mutation** ***BRCA2* :c.1300_1303del** **by Sanger sequencing**

Patient BC01


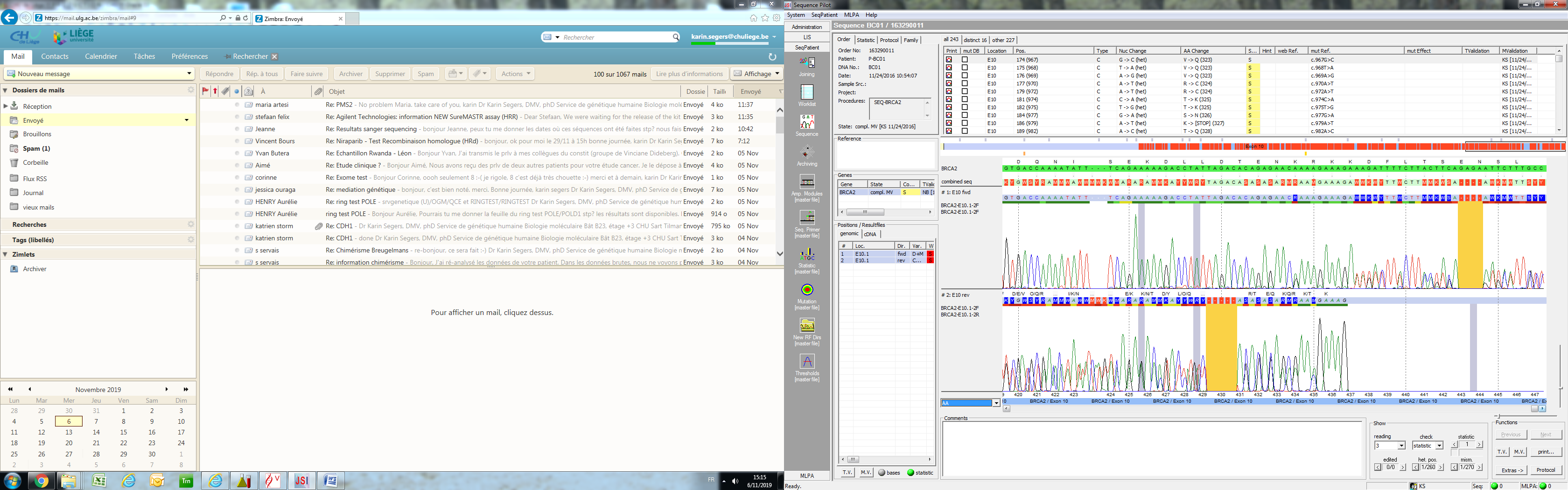


**Supplementary data S3. Confirmation of pathogenic mutation** ***BRCA1*:c.4065_4068del by Sanger sequencing**

Patient BC022


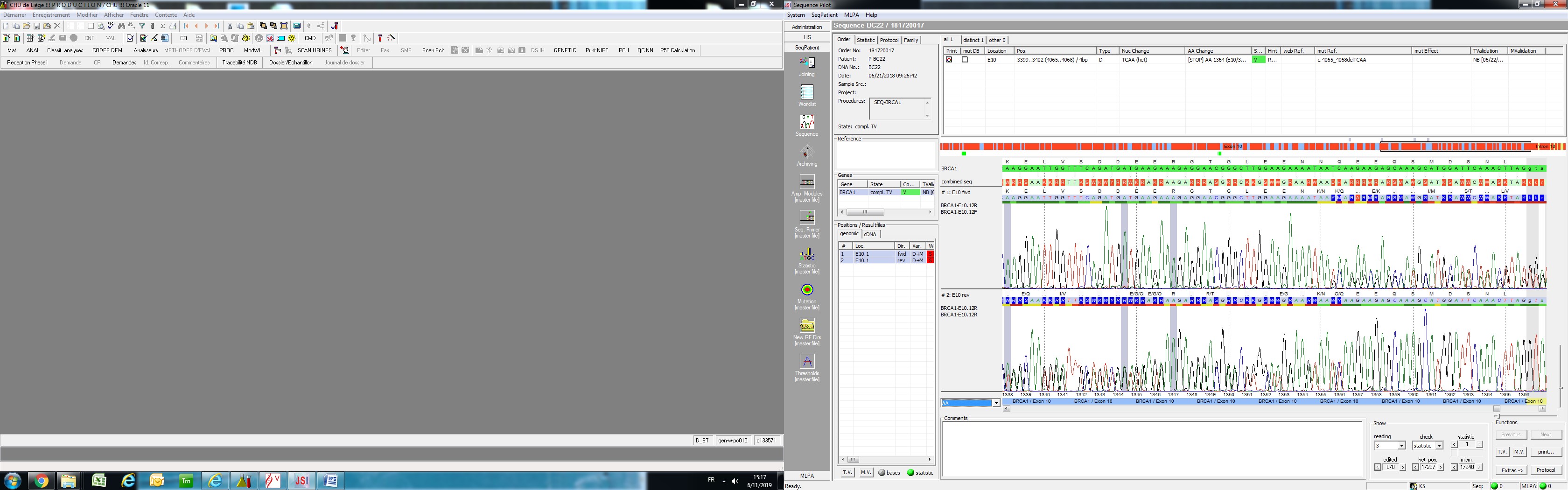


**Supplementary data S4. Confirmation of likely pathogenic mutation** ***TP53* :c.726C>G** **by Sanger sequencing**

Patient BC023


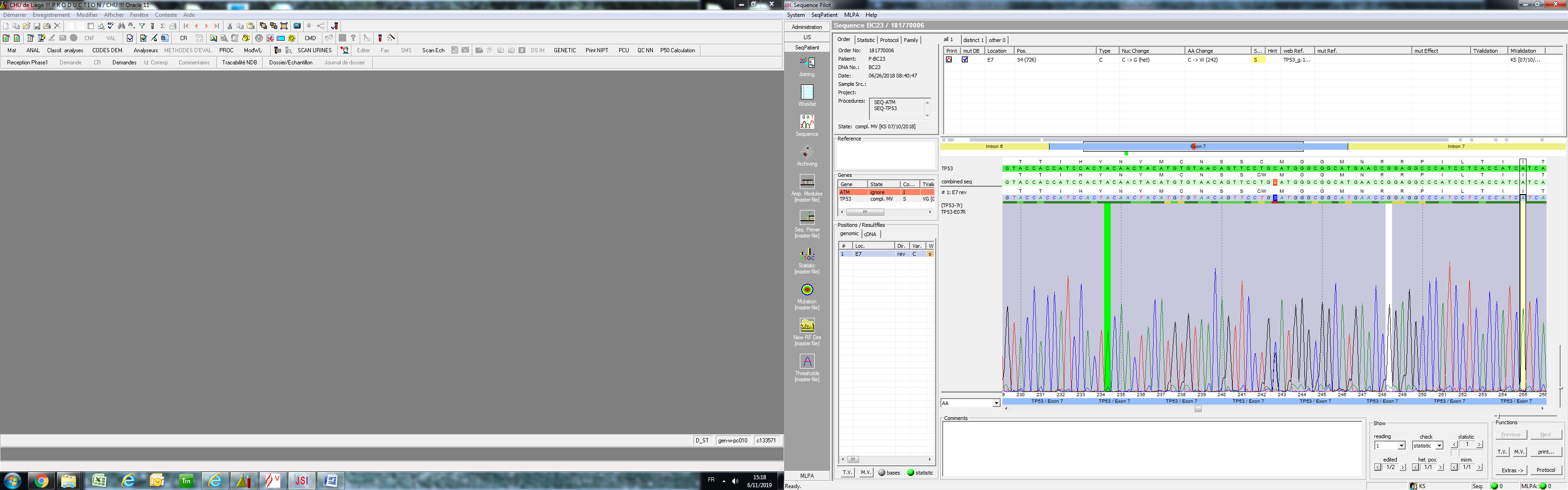


**Supplementary data S5. Confirmation of pathogenic mutation** ***BRCA2*:c.9096dupA by Sanger sequencing**

Patient BC40


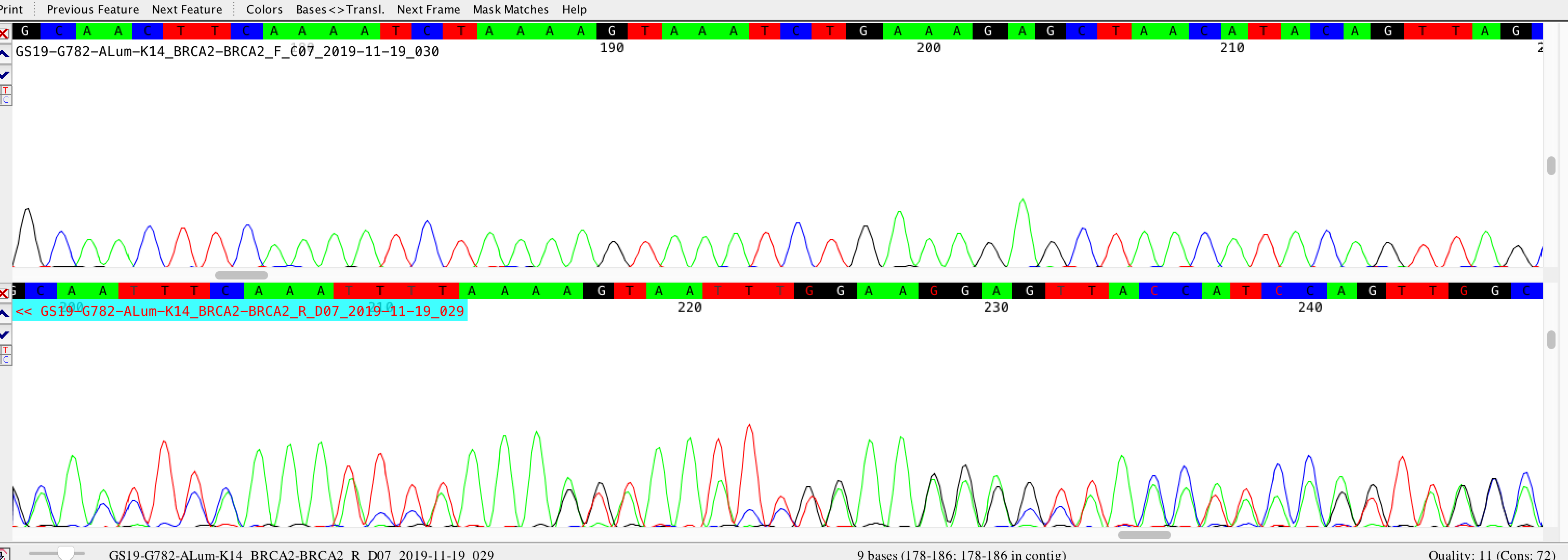

Supplement: Supplementary file 1 — Supplementary Material [file MGG3-8-e1500-s001.doc]
